# Adult Premenopausal Bone Health Related to Reproductive Characteristics—Population-Based Data from the Canadian Multicentre Osteoporosis Study (CaM*os*)

**DOI:** 10.3390/ijerph15051023

**Published:** 2018-05-18

**Authors:** Azita Goshtasebi, Claudie Berger, Susan I. Barr, Christopher S. Kovacs, Tanveer Towheed, K. Shawn Davison, Jerilynn C. Prior

**Affiliations:** 1 Centre for Menstrual Cycle and Ovulation Research; University of British Columbia, Vancouver, BC V5Z 1M9, Canada; azita.goshtasebi@ubc.ca (A.G.); susan.barr@ubc.ca (S.I.B.); 2 Canadian Multicentre Osteoporosis Study, Research Institute of the McGill University Health Centre, Montreal, QC H4A 3S5, Canada; claudie.berger@mail.mcgill.ca; 3 Food, Nutrition and Health Program, University of British Columbia, Vancouver, BC V6T 1Z4, Canada; 4 Medicine (Endocrinology and Metabolism), Obstetrics & Gynecology, and BioMedical Sciences, Memorial University of Newfoundland, St. John’s, NL A1B 3V6, Canada; ckovacs@mun.ca; 5 Medicine and Public Health, Queens University, Kingston, ON, K7L 3N6 Canada; tt5@queensu.ca; 6 A Priori Medical Sciences Inc., Victoria, BC V8R 3E3 Canada; ebmedicine@gmail.com; 7 Department of Medicine, Division of Endocrinology and Metabolism, University of British Columbia, Vancouver, BC V5Z 1M9, Canada; 8 School of Population and Public Health, University of British Columbia, Vancouver, BC V6T 1Z4, Canada; 9 BC Women’s Health Research Institute, Vancouver, BC V6H 2N9, Canada

**Keywords:** menstrual cycle, menarche age, androgen excess, amenorrhea, oligomenorrhea, infertility, parity, population-based, bone mineral density, prevalent fragility fracture

## Abstract

Amenorrhea is important for women’s bone health. However, few have reported reproductive, anthropometric (body mass index [BMI], height) and bone health (areal bone mineral density [BMD], prevalent fractures) in a population-based study. The purposes of this cross-sectional study of women in the randomly-selected Canadian Multicentre Osteoporosis Study (CaM*os*) population were: (1) to describe reproductive, demographic, anthropometric and lifestyle variables; and (2) in menstruating women, to relate reproductive and other variables to BMD at the lumbar spine (L1-4, LS), femoral neck (FN) and total hip (TH) sites and to prevalent fragility fractures. This study describes the reproductive characteristics of 1532 women aged 30–60 years. BMD relationships with reproductive and other variables were described in the 499 menstruating women. Mean menarche age was 12.8 years, 96% of women were parous and 95% had used combined hormonal contraceptives (CHC). Infertility was reported by 9%, androgen excess by 13%, amenorrhea by 8% and nulliparity by 4%. LS BMD was negatively associated with amenorrhea and androgen excess and positively related to current BMI and height. A later age at menarche negatively related to FN BMD. BMI and height were strongly related to BMD at all sites. Prevalent fragility fractures were significantly associated with quartiles of both LS and TH BMD.

## 1. Introduction

A few excellent population-based studies (meaning a random sample from an entire population) have described reproductive characteristics in adolescent [1], mixed adolescent-adult [2] and premenopausal [3] cohorts identifying menarche age, the proportion with amenorrhea, oligomenorrhea and infertility as well as parity, lactation and reproductive surgeries. Reproductive and anthropometric variables, however, are complexly interrelated. For example, earlier age at menarche in Canadian population data was found to relate to a higher risk for adult obesity [4]; it has also been associated with higher mortality [5]. Within normal and overweight ranges, a higher body mass index (BMI; weight in kg/height in m^2^) is one of the most important associations with a higher areal bone mineral density (BMD; gram/cm^2^) and perhaps also in preventing fractures [6]. However, when BMI increases into the obese range (BMI ≥ 30) the fracture risk was found to be significantly *increased*, based on BMD-adjusted international data from large epidemiological cohorts [7]. Thus there are complex interrelationships among reproductive and body weight/height variables, BMD and fracture risk.

Some epidemiological studies have related women’s reproduction to BMD or to incident fractures [8]. For example, in the Iowa Women’s Health Study (random sample, 42% participation rate), recall of both past “irregular” cycles plus variable durations of menstrual flow by elderly menopausal women was associated with an 82% higher risk for incident hip fracture after adjusting for BMI, hysterectomy and ovariectomy [8]. At present, however, it is not clear what combination of reproductive, anthropometric, demographic and lifestyle variables is related to lower BMD values and higher prevalent fracture risks in population-based data. Therefore, the purpose of this study in the population-based premenopausal adult Canadian Multicentre Osteoporosis Study (CaM*os*) population was to describe these reproductive and anthropomorphic variables and their relationships with BMD levels and with prevalent fragility fractures.

## 2. Materials and Methods

### 2.1. Participants

The participants were women in the population-based CaM*os* cohort. The design, objectives and methods of CaM*os* have previously been described [9]. Briefly, the study recruited community dwelling participants aged 25–80+ who lived within a 50-km radius of one of nine Canadian cities (St John’s, Halifax, Quebec City, Toronto, Hamilton, Kingston, Saskatoon, Calgary and Vancouver) and could converse in English, French (Quebec City only) or Chinese (in Vancouver and Toronto only). Households were randomly selected from residential phone numbers; participants were then randomly selected within households by a sex and age-stratified protocol weighted to older adults and targeting two-thirds women. Of those randomly selected, 42% agreed to full participation including questionnaires plus clinical measurements of height and weight (used to calculate BMI), BMD and spine radiographs; participation rates were higher in women and younger participants (data not shown).

Ethics approval was granted through McGill University (#94-07-19 RECt) and all centres’ ethics review boards. All participants gave written informed consent and the study is conducted in accordance with the Helsinki Declaration. 

### 2.2. Flow of Participants through the Study

**Figure 1:**
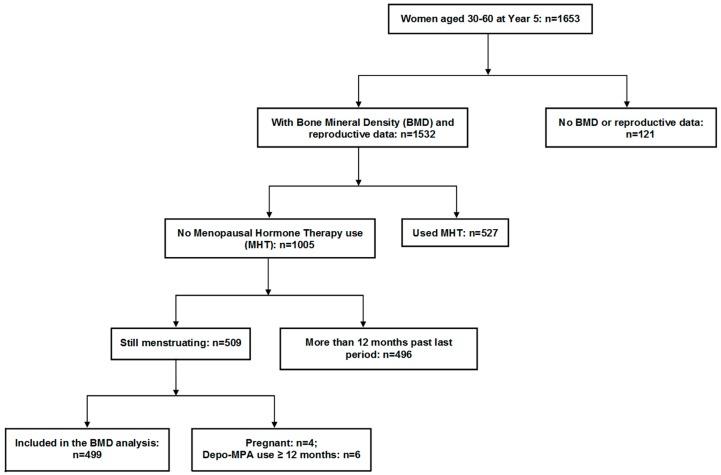
Diagram showing the flow of participants through this observational, cross-sectional study of reproductive, anthropomorphic and bone health variables in younger women in the Canadian Multicentre Osteoporosis Study.

This study includes full cohort examination at baseline and Year five (Y5) plus data from those at Y3 who at baseline had been ages 40–60 years (Figure 1). For the purposes of description of reproductive variables, we included data from women aged 30–60 years at the Year 5 (Y5) examination for two reasons: (1) the questions about infertility and androgen excess were first included for all women in the Y3 questionnaire that did not include data for all women; and (2) baseline reproductive data had previously been reported as confounding variables for a cross-sectional study of combined hormonal contraceptives (CHC) and BMD [10].

For analysis of the relationships of reproductive, anthropometric and other variables with BMD and prevalent fracture, we included all pre- or perimenopausal women who, at Y5 were ages 30–60 years, who were still menstruating or had not had bilateral ovariectomy and who were not pregnant or had not been on depot-medroxyprogesterone for longer than a year (Figure 1).

### 2.3. Methods 

As described previously [9], the data were collected using an interviewer-administered questionnaire at baseline, Y3 (ages 40–60 years at baseline) and Y5 for each age-appropriate women. We extracted demographic, anthropometric, nutritional and lifestyle as well as reproductive information from all three interviews and examinations.

Age at menarche was the age at first menstruation; it was extracted from the baseline questionnaire; gynecological age was defined as the current age minus menarche age. Those with early menarche (≤10 years) and late menarche (≥16 years) were also tabulated. Parous women reported at least one live birth—parity was reported as total number of lifetime live births. Months of lactation (breast feeding) were totaled related to all children. Nulliparity (no live births), parity and lactation were derived from all three questionnaires. History of ever experiencing oligomenorrhea or amenorrhea was extracted from questionnaire data in all three interviews. Oligomenorrhea was reported when women said, at any of interviews, that they had ever experienced cycle intervals longer than 35 days but less than 90 days. Amenorrhea was reported when women said that they had ever had a cycle interval longer than 90 days at any of interviews. Androgen excess was defined as ever experiencing unwanted facial hair (hirsutism) or acne that needed medical attention (from Y3 and Y5 questionnaires: “*Have you ever been sufficiently bothered by severe acne, unwanted face or body hair to consult a physician for treatment?*”). Infertility was defined as being partnered and unsuccessful at achieving pregnancy after a year or more. Women reported infertility was due to “hormonal” variables if they had anovulatory androgen excess (AAE/also known as polycystic ovarian syndrome, PCOS) or hypothalamic ovulatory disturbances (anovulation or short luteal phases commonly occurring silently in regular cycles) [3]. If infertility were due to anatomical blockage of the fallopian tubes, it was termed “obstructive” and if it were related to the man in the partnership, it was considered “male partner-related”. Infertility data were extracted from Y3 and Y5 questionnaires. CHC (in oral, vaginal ring or patch formats) was considered to have been used when women reported they had taken it for three or more months [10]. 

Demographic variables such as age and race were obtained from the baseline interview. There were likely fewer non-Caucasian participants than their proportion in the population since 11% of Canadians in 1996 were “visible minorities” [11]. Height and weight were recorded at each interview and were used to calculate BM1. At baseline, a woman’s reported tallest height and recalled weight when she was 18 years old were used to calculate her BMI at age 18. Weight cycling was defined as one or more episodes of losing and regaining more than 10 pounds (4.5 kg).

The average duration and number of cigarettes smoked were transformed into an “ever smoked” variable for those smoking more than 10 cigarettes a day for more than six months. Alcohol consumption was reported as average number of standard-sized alcoholic beverages per week. Exercise was reported as average daily kilocalories (Kcal) burned during vigorous, strenuous and moderate exercise/week. Education was reported as having a “professional certificate” if participants had two years of post-high school study/training. 

Interpreting alcohol, Vitamin D and calcium intakes at Y5 was problematic because all women at baseline had been informed of their BMD results [12]; some women appeared to have changed their baseline behaviors in response to knowing their BMD, and those with low BMD values appeared to be more likely to have done so (data not shown). Therefore, Y0 (baseline data) were used to calculate the average total daily intakes of calcium and Vitamin D based on an abbreviated semi-quantitative standardized food frequency questionnaire and reported supplements.

Seven centers had Hologic densitometers and two centers had Lunar densitometers. All Lunar measurements were converted to equivalent Hologic values using standard reference formulas [13]. Quality control was assured, calibration between centers was accomplished and longitudinal drift was assessed; these important variables were assessed and all data were integrated using a common anthropomorphic BMD phantom as detailed elsewhere [14,15].

For the assessment of bone health, we included Y5 calibrated [14] BMD measured by dual energy X-ray absorptiometry (DXA) for the lumbar spine (LS, segments L1-L4), femoral neck (FN) and total hip (TH) in the analysis. We also included all women who reported one or more prevalent fragility fractures; these could have been reported at baseline or between baseline and Y5. They all involved a force equivalent to a fall from a standing height or less (excluding fractures of the head, hands and feet as is conventional in osteoporosis assessments).

### 2.4. Statistical Analysis

Means and standard deviations (SDs) were used for continuous variables and proportions for categorical variables to describe the sample in terms of reproductive, sociodemographic, anthropometric and lifestyle characteristics. A Pearson correlation matrix of all independent continuous variables for each of three BMD sites was generated to assess possible correlations. A T-test was carried out to assess BMD relationships with continuous variables, and the Chi square test facilitated evaluation of the association between categorical variables and BMD values. Analysis of Variance (ANOVA) was used to assess differences in continuous variables across BMD quartiles. For percentage of participants with any prevalent fragility fracture, we used Chi Square analysis to show any trends across quartiles of L1-4 spine BMD. For percentage with any prevalent fragility fracture and to assess whether there were BMD trends among those with lifetime normal (regular, normal length) menstrual cycles and those who ever experienced oligo- or amenorrhea, we also used the Chi Square test.

Linear regression was used to assess the relationships among BMD and variables that showed a statistically significant association with BMD in correlation and T-test analyses for each BMD site. Categorical values that had the strongest relationship for the BMD within each group of variables were added one by one to the model at each step. The categories were added in the following order and included the most strongly related of variables from demographics (age, race), anthropometric and physical activity data (current BMI at Y5, height at Y5, exercise Kcal) and reproductive (age at menarche, CHC use, live births, breast feeding, oligomenorrhea, amenorrhea and androgen excess) variables. Parameter estimates with their 95% confidence intervals (CI) as well as the coefficient of determination (R^2^) were reported. SPSS software (IBM Corp. Released 2016, IBM SPSS Statistics for Windows, Version 24.0, Armonk, NY, USA) was used for all analyses.

## 3. Results

This study included 1653 women from the national adult CaM*os* cohort ages 30–60 at Y5 of this prospective, observational population-based study. As shown in Figure 1, fewer women had complete reproductive data (*n* = 1532). For comparing reproductive and other variables with bone health parameters (BMD and prevalent fragility fractures), we examined data from the 499 women who were still menstruating and were not excluded due to pregnancy or injections of medroxyprogesterone for contraception.

### 3.1. Reproductive Characteristics

The reproductive characteristics of all women aged 30–60 in the CaM*os* cohort at Y5 with these data are included in Table 1. In the 1532 women for whom all reproductive data were available, menarche occurred at an average age of 12.8 years with 5.9% having an early menarche at ≤ age 10; five percent had a late menarche at age 16 or older. In adolescence, 9.1% of women reported that they needed medication to achieve regular menstruation following menarche. Androgen excess for which they sought medical therapy was reported by 13%. Ever experiencing amenorrhea occurred for eight percent of women; 10 percent had a history of ever experiencing oligomenorrhea. Four percent (4.4%) of this cohort were nulliparous and nine percent (9.4%) reported infertility. Parous women on average had 2.2 live births and breast fed for an average of seven months (total for all infants). CHC use was reported for at least three months by 95.3%. Other reproductive characteristics of the primary study sample are also shown in Table 1.

### 3.2. Relationship of Reproductive Variables with Bone Health (BMD, Prevalent Fracture)

As shown in Figure 1, a total of 499 women were pre- or perimenopausal and met inclusion criteria for the analysis of the relationship of reproductive and anthropometric variables with bone health. Baseline characteristics of the study sample by LS BMD quartiles at Y5 are presented in Table 2. The included women were mostly white (94.1%), 72% had secondary education plus some university (in other words, a “professional certificate”), and their average BMI was in the overweight range (BMI: 26.3 kg/m^2^). The mean calories burned with exercise, smoking and alcohol consumption, and total intakes of calcium and Vitamin D did not differ between LS BMD quartiles.

A Bonferroni post hoc test of pair-wise, quartile differences across the four LS BMD quartiles revealed that these were statistically significant only for the weight, height and BMI variables (*p* values all <0.01). Age at menarche tended to decrease (*p* = 0.07) across LS quartiles but it was not linear. Parity was not related to LS BMD quartiles, but the percent of women who had ever breastfed for more than six months increased from LS quartiles 1 to 3 (*p* = 0.037). Both the FN and TH showed linear increases with the increasing quartiles of LS BMD. Evaluation of reproductive variables and their associations with FN and TH BMD quartiles indicated that only menarche age was related; older ages at menarche tended to occur in the lowest quartiles of hip BMD variables (data not shown).

The occurrence of a prevalent (past) fragility fracture, collected at baseline as well as between Y0 and Y5, differed significantly across the four LS quartiles by ANOVA, although the trend did not appear to be linear. The percentage of women with prevalent fragility fractures across quartiles of FN was not significant according to ANOVA, with 18%, 17%, 8% and 11%, respectively, from lowest to highest BMD quartiles (*p* = 0.08) (data not shown). For TH BMD, the prevalences of fragility fractures were 19%, 12%, 17% and 6% for quartiles one through four, respectively (*p* = 0.03) (data not shown).

**Figure 2:**
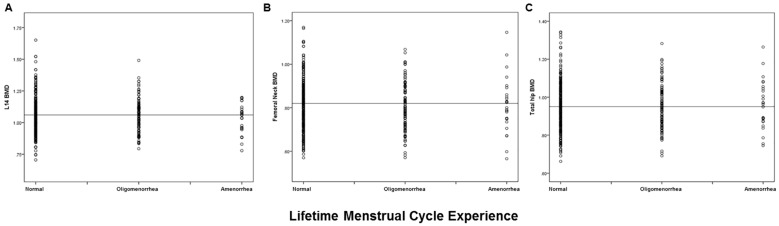
Scatter Plots of areal Bone Mineral Density at three sites by lifetime Menstrual Cycle Experience in women ages 30–60 in the Canadian Multicentre Osteoporosis Study. ANOVA was used to evaluate whether there was a trend across the menstrual cycle experiences by site. (**A**): Lumbar Spine (L1-4) (*p* = 0.68), (**B**): Femoral Neck (*p* = 0.61) and (**C**): Total Hip (*p* = 0.85) sites. The horizontal line represents the mean value for BMD in the normally cycling women.

Figure 2 presents a comparison of BMD distributions by reported lifetime menstrual cycle characteristics (regular, ever experiencing oligomenorrhea or ever experiencing amenorrhea) at each of the three BMD sites. There was no statistically significant difference in mean BMD among these types of disturbed cycle lengths versus normal menstruation for any of the three BMD sites. ANOVA results indicated there was also no trend toward decreased BMD with a history of ever experiencing amenorrhea.

The adjusted associations by linear regression among reproductive characteristics, covariates and BMD values for each site are presented in Table 3. Experiencing amenorrhea and androgen excess were both negatively related to lumbar spine BMD. Age at menarche was negatively associated with femoral neck BMD. Current BMI and height were associated with BMD at all three bone sites. The total variance explained by all variables (R^2^) and the additions to R^2^ from each included variable are shown below the table.

The order of categories of variable’s entry into the model (Table 3) is described in Methods. The variables were: Age: continuous; Race: other, Caucasian; Exercise energy expenditure: continuous (only for FN BMD as it was too highly correlated with L1-4 and TH to include); Current BMI: continuous; Current height: continuous; Age at menarche: continuous; Years of CHC use: continuous; Months of breast feeding: continuous; Number of live births: continuous; History of amenorrhea: no, yes; History of oligomenorrhea: no, yes; History of medical care for androgen excess: no, yes.

Eighteen percent of LS BMD was explained (R^2^ = 0.179) by positive contributions of current BMI and height and negative influences from an amenorrhea and from an important androgen excess history. The femoral neck BMD (R^2^ = 0.209) was positively accounted for by current BMI and height and negatively related to age at menarche. For the TH BMD (R^2^ = 0.232), the only contributing variables were current BMI and height. Thus, reproductive characteristics negatively contributed to the lumbar spine and femoral neck BMD sites, whereas anthropometric variables were importantly related to BMD at all three sites.

## 4. Discussion

This cross-sectional, population-based, Canada-wide study of 499 menstruating women ages 30+ years found that a history of amenorrhea (although in only 8%) and of androgen excess (in 17%) of all women negatively contributed to LS BMD values. Age at menarche was negatively related to FN BMD. However, as expected, all three BMD sites were positively related to body mass index (BMI) [6] that accounted for 63%, 73% and 88% of the explained variance at L1-4, FN and TH sites respectively. Prevalent fragility fractures were reported by significantly more women in the lower LS BMD quartiles [1,2,3] compared with the highest BMD quartiles. There was also a significant relationship between prevalent fractures and TH BMD quartiles. To our knowledge, this is the first observational study that has ever reported that androgen excess *at a population level* (in an analysis including BMI and height as well as amenorrhea) is related to *lower* spinal BMD values. However, androgen excess was not significantly related to quartiles of LS BMD.

Androgen excess, commonly associated with polycystic ovary syndrome (PCOS), but also called Anovulatory Androgen Excess (AAE) [16], is believed to cause an *increase* in BMD based on multiple studies [17,18]. However, newer information has suggested that these higher BMD values depend on having a more *regular* cycle, since women with PCOS and oligomenorrhea had similar BMD values as controls [19]. In PCOS/AAE, BMD has also been strongly related to BMI which tends toward obesity [18]. However, women with PCOS/AAE are relatively, or absolutely, deficient in progesterone due to frequent ovulatory as well as cycle disturbances. Lower progesterone levels related to ovulatory disturbances were associated with significant bone loss in women without PCOS/AAE [20,21]. This new observation requires further investigation.

Amenorrhea occurring after menarche was present in 8% of this cohort but did not differ across quartiles of LS BMD (Table 2) nor from the BMD of regularly cycling women at the LS, FN, or TH sites as shown in Figure 2. However, it was associated with lower LS BMD in a model including BMI and height as well as androgen excess. A large data-set in 30–39 year-old Chinese women who were predominantly farmers showed similar negative BMD relationships with amenorrhea, but at the FN and TH sites, rather than the LS [22]. One difference may be that these Chinese data examined spinal segments L2-4 rather than L1-4 which tends to be more sensitive to change and that their lumbar spine model explained very little of the variance. The Michigan Women’s Bone Health Study, also a population-based cohort, documented that women reporting “irregular” cycles (which were defined as oligomenorrhea or amenorrhea) had different rates of BMD change than those with regular cycles [23]. However, because they did not differentiate women with and without androgen excess, there were some women whose BMD changes were positive and some for whom they were negative [23]. 

Later age at menarche was negatively related to femoral neck BMD in a linear regression and was borderline associated with lower quartiles of LS in these data. These results confirm other publications reporting that a later age at menarche was associated with a lower trochanter BMD in Chinese from Hong Kong [24] as well as with lower forearm BMD in population-based data from the mid-Norway HUNT study [25] and from teens in the northern Norway “Fit Futures” study [26]. A similar association has been shown in a prospective study of adolescents from France [27].

Parity was protective of incident fragility fractures as was longer reproductive lifespan (40 versus 30 years) in the population-based Dubbo Australian cohort of women with a mean age of 70 years [28]. They also showed that nulliparous compared with parous menopausal women had significantly lower BMD values [28]. Similar results were documented in the population-based Norwegian HUNT study related to forearm BMD in menopausal women [29]. In this study, parity and nulliparity were not associated with spine BMD, but a history of breast feeding was positively related to the first three of LS quartiles in these menstruating women. A similar positive relationship has previously been reported [30]. In the large and prospective but not population-based cohort of the USA’s Women’s Health Initiative observational data, parity was not and breast feeding only weakly related to incident hip and other fractures [31]. We were unable to relate bone health variables to reproductive lifespan in the still-menstruating women in this cohort in whom we assessed bone health; they were not yet menopausal. 

Prevalent fractures are rarely described related to BMD in population-based data. These data showed significant relationships of more fragility fractures in lower quartiles of BMD at the LS (non-linear) and overall with the TH. Some authors have shown that prevalent fractures in peri-pubertal girls are strongly related to previous fracture, age and bone measurements [32]. A prospective study of pre-peripubertal girls also documented that that a later age at menarche was strongly related to lower forearm [27] and tibial [33] volumetric bone mineral density by high resolution peripheral computed tomography. 

Although weight cycling has been associated with lower BMD values in women [34], it was more prevalent in women in the highest LS quartile in this population in parallel with higher BMI values. This is likely explained by the higher weight gained since age 18 years in this quartile given the tendency for those with more episodes of weight cycling to be heavier and the strong influence of BMI on BMD. In population data in both women and men, weight cycling has been related to an increased risk of hip fracture [35]. Cognitive dietary restraint may also have entered these BMD models [36] but the pertinent questions were not asked at the Y5 interview whose data this study examined. 

These data are limited by their cross-sectional nature. In addition, we could not assess the relationships of lifetime reproductive duration [37,38] with bone health measures because the BMD and prevalent fracture assessments we performed were in menstruating women who were still pre- or perimenopausal.

This study also has several strengths. These include that: (1) it studied a random sample from a national population and therefore is representative of 41% of the Canadian population [9]; (2) it included a broad and comprehensive interviewer-administered questionnaire that acquired a broad assessment of pertinent reproduction information; and (3) evaluations of bone were completed with calibrated areal BMD measurements at three different commonly clinically obtained proximal sites, and (4) we also assessed the prevalence of one or more fragility fractures related to BMD quartiles.

## 5. Conclusions

This cross-sectional study of reproduction related to bone health in the Canadian Multicentre Osteoporosis Study assessed both bone mineral density (BMD) and prevalent fragility fractures in almost 500 still-menstruating women. We confirmed previous positive associations of BMI and height with higher BMD and of lower BMD with later ages at menarche. We also showed, as have others, that ever experiencing amenorrhea is negatively related to spine BMD when BMI and height are in the model. In addition, in a lumbar spine model including BMI, height and amenorrhea, we discovered for the first time that androgen excess was related to lower lumbar spine BMD. We further showed that lower lumbar spine and total hip BMD values were associated with the experience of past fragility fracture. Prospective research is required to determine whether these reproductive variables relate to lower BMD in late perimenopause and thus, potentially, to higher risks for *incident* fragility fractures later in life.

## Figures and Tables

**Table 1:** Reproductive characteristics of women aged 30–60 years in the population-based Canadian Multicentre Osteoporosis Study.

Characteristic	Mean (SD/%)	*n*
Age, years	51.48 (7.43)	1653
Age at menarche, years	12.75 (1.54)	1646
≤10 years at menarche, *n* (%)	97 (5.9)	
≥16 years at menarche, *n* (%)	84 (5.1)	
Regular menses after menarche, *n* (%)		
Immediately	1318 (79.8)	1651
Became regular spontaneously	64 (19.5)	329
Became regular with medication	151 (56)	268
Parity, *n* (%)		1403
Nulliparous (0 births)	62 (4.4)	
1–2 births	874 (62.3)	
3 or more births	467 (31.1)	
^1^ Breast feeding, *n* (%) months		1328
Never	496 (37.3)	
≤6 months	367 (27.6)	
>6 months	465 (35)	
^1^ CHC use, *n* (%) duration		1390
≤3 months = “never”	66 (4.7)	
4–12 months	215 (15.5)	
>12 months	1109 (79.8)	
^2^ Oligomenorrhea, yes, *n* (%)	164 (10.1)	1505
^3^ Amenorrhea, yes, *n* (%)	137 (8.3)	1516
Infertility, *n* (%)	145 (8.8)	1653
Hormonal	45 (2.7)	
Anatomical, obstructive	37 (2.2)	
Male factor	15 (0.9)	
^4^ Other	48 (2.9)	
Androgen Excess, yes, *n* (%)	199 (13.0)	1653
Hysterectomy, yes, *n* (%)	393 (23.8)	1653
Ovariectomy, *n* (%)	230 (13.9)	1653
Unilateral	95 (5.7)	
Bilateral	130 (7.9)	
Unsure	5 (0.3)	
Natural menopause, yes, *n* (%)	532 (43.7)	1217
Age at natural menopause, years	50.6 (4.1)	
Reproductive lifespan, years	38.7 (7.6)	

^1^ CHC = combined hormonal contraception (could be as a pill, patch or vaginal ring) used for ≥3 months; ^2^ If a women recorded her periods as >35 to 89 days apart at any time; ^3^ If a women reported her periods as ≥90 days apart at any time; ^4^ Including undetermined, uterine structural disorders, multiple factors.

**Table 2:** Demographic, nutritional and reproductive characteristics of pre-perimenopausal population-based women in the Canadian Multicentre Osteoporosis Study (year 5, *n* = 499) by quartiles of lumbar spine BMD (L1-4). Those that show statistical significance (*p* of ≤ 0.05) are in **bold.**

Characteristic *	Overall Mean (SD)	*n*	Lumbar Spine (L1-4) BMD Quartiles				*p* **
			1	2	3	4	
L1-4 BMD mean (SD), g/cm^2^	1.053 (0.135)	462	0.891 (0.050) *n*:115	1.005 (0.026) *n*:115	1.088 (0.024) *n*:116	1.228 (0.096) *n*:116	–
Range of LS (L1-4) values g/cm^2^	0.71 to 1.65		0.71 to 0.95	0.96 to 1.04	1.05 to 1.12	1.13 to 1.65	–
Mean age (SD), years	43.7 (6.9)	462	44.1 (6.8) *n*:115	43.6 (7.2) *n*:115	44.2 (6.6) *n*:116	43.1 (7.0) *n*:116	0.59
Mean current height (SD), cm	162.4 (6.5)	425	160.5 (6.5) *n*: 102	161.6 (6.7) *n*:105	163.2(6.3) *n*:106	164.3 (6.1) *n*:112	**0.001**
Mean current weight (SD), kg	69.5 (15.4)	424	60.9 (11.8) *n*:102	67.4 (12.8) *n*:105	71.8 (14.3) *n*:106	77.2 (17.3) *n*:111	**0.001**
Mean current BMI (SD), kg/m^2^	26.31 (5.52)	424	23.65 (4.51) *n*:102	25.79 (4.55) *n*:105	26.97 (5.36) *n*:106	28.06 (6.25) *n*:111	**0.001**
Mean BMI at age 18 (SD), kg/m^2^	21.06 (3.10)	433	20.15 (2.69) N:110	20.56 (2.69) *n*:108	21.54 (3.37) *n*:106	21.99 (3.26) *n*:109	**0.001**
Mean BMI change from age 18 (SD), kg/m^2^	5.25 (4.59)	399	3.58 (4.20) *n*:99	5.20 (3.97) *n*:99	5.62 (4.31) *n*:97	6.54 (5.28) *n*:104	**0.001**
Race (%), white	94.2	499	92.2	91.3	94.8	98.3	0.10
Education (% yes) ≥ professional certif.	71.9	499	72.2	76.5	71.6	67.2	0.48
* Ever smoked (% yes)	45.0	499	40.9	38.3	45.7	55.2	0.051
Mean alcohol use (SD), drink/week	2.4 (3.6)	462	1.9 (2.9) *n*:115	2.5 (4.2) *n*:115	2.8 (4.6) *n*:116	2.4 (3.7) *n*:116	0.61
* Exercise mean (SD) expenditure Kjoules/week	4627 (3582)	451	4051 (3423) *n*:111	4482 (3616) *n*:112	4842 (3706) *n*:112	5111 (3536) *n*:116	0.13
* Calcium—mean intake (SD), mg/d	955 (572)	446	905 (620) *n*:111	999 (587) *n*:111	984 (630) *n*:110	935 (435) N:114	0.58
* Vitamin D mean intake (SD), µg/d	4.7 (5.5)	457	4.7 (6.3) *n*: 113	5.2 (5.5) *n*: 114	4.5 (4.7) *n*: 114	4.4 (5.5) *n*: 116	0.71
* Weight cycling (%), yes	30.3		26.1	23.5	29.3	42.2	**0.005**
Age at menarche mean (SD), years	12.8 (1.4)	459	13.1 (1.6) *n*:114	12.7 (1.5) *n*:113	12.7 (1.2) *n*:116	12.6 (1.5) *n*:116	0.07
* Gynecological age mean (SD), years	31.0 (7.1)	459	31.1 (7.1) *n*:114	30.9 (7.5) *n*:113	31.5 (6.8) *n*:116	30.5 (6.9) *n*:116	0.40
* Parous (%), yes	94.3	395	94.5	97.8	95.9	89	0.16
* Ever used CHC (%), yes	85.3	443	85.2	86.1	86.2	83.6	0.94
* Ever Breastfed (%), yes	37.7	301	27.0	40.0	44.8	38.8	**0.037**
* Androgen excess (%), yes	16.7	499	22.6	14.8	11.2	18.2	0.11
* Oligomenorrhea (%), yes	24.9	499	23.5	23.5	28.4	24.1	0.78
* Amenorrhea (%), yes	7.6	499	11.3	5.2	7.8	6.1	0.31
Femoral neck BMD (SD), g/cm^2^	0.810 (0.112)	449	0.673 (0.042) *n*:112	0.769 (0.022) *n*: 112	0.842 (0.021) *n*:113	0.958 (0.066) *n*:113	−
Total hip BMD mean (SD), g/cm^2^	0.951 (0.122)	444	0.804 (0.048) *n*:111	0.902 (0.025) *n*:111	0.987 (0.027) *n*:111	1.110 (0.122) *n*:111	−
Women with any Prevalent Fragility Fracture, *n* (%)	62 (13.5)	499	20 (17.5)	14 (12.2)	21(18.3)	7 (6.1)	**0.03**

* See methods for details on the variables. ** *p* value for differences across the four quartiles of BMD is derived using analysis of variance (ANOVA) for continuous variables and by Chi-Square for variables expressed as percentages.

**Table 3:** Linear Regression Results by each areal Bone Mineral Density site in pre- and perimenopausal women in the Canadian Multicentre Osteoporosis Study.

Estimate (95% CI)	Lumbar Spine (*n* = 423) g/cm^2^	Femoral Neck (*n* = 409) g/cm^2^	Total Hip (*n* = 407) g/cm^2^
Current BMI (kg/m^2^)	0.009 (0.006 to 0.012)	0.008 (0.05 to 0.010)	0.010 (0.008 to 0.013)
Current Height (cm)	0.004 (0.001 to 0.006)	0.004 (0.002 to 0.006)	0.003 (0.001 to 0.005)
Menarche age (years)		−0.011 (−0.020 to −0.001)	
Amenorrhea (yes)	−0.079 (−0.141 to −0.018)		
Androgen excess (yes)	−0.050 (−0.092 to −0.008)		
**Total R^2^**	**0.176**	**0.209**	**0.232**
Model 1—BMI: R^2^ = 0.111		Model 1—BMI: R^2^ = 0.153	Model 1—BMI: R^2^ = 0.205
Model 2—height added to R^2^ 0.025 Model 3—amenorrhea added to R^2^ 0.021		Model 2—height added to R^2^ 0.039 Model 3—age at menarche added to R^2^ 0.017	Model 2—height added to R^2^ 0.027
Model 4—androgen excess added to R^2^ 0.019			

## Abbreviations

AAE	Anovulatory Androgen Excess
ANOVA	Analysis of variance
BMD	areal Bone Mineral Density
BMI	Body Mass Index
CaM*os*	Canadian Multicentre Osteoporosis Study
CeMCOR	Centre for Menstrual Cycle and Ovulation Research
CHC	Combined Hormonal Contraceptives
FN	Femoral Neck
LS	Lumbar Spine
PCOS	Polycystic Ovary Syndrome
TH	Total Hip
